# The need for a disaster readiness mindset: A key lesson from the coronavirus disease 2019 (COVID-19) pandemic

**DOI:** 10.1017/ice.2021.26

**Published:** 2021-01-25

**Authors:** Zhaohui Su, Dean McDonnell, Junaid Ahmad

**Affiliations:** 1 Center for Smart and Connected Health Technologies, Mays Cancer Center, School of Nursing, UT Health San Antonio, San Antonio, Texas, United States; 2 Department of Humanities, Institute of Technology Carlow, Carlow, Ireland; 3 Prime Institute of Public Health, Peshawar Medical College, Peshawar, Warsak Road, Peshawar, Pakistan

*To the Editor*—The world at large lacks disaster readiness. The history of humanity includes narratives of how we have faced and overcome challenges and difficulties, both natural and human made, both large and small.^1,2^ In parallel to a racial pandemic,^3^ coronavirus disease 2019 (COVID-19) has also been linked to social inequality in its many forms and is another test of humanity’s resolve.^4^ Despite the depth of knowledge and experience with challenges that humanity has accumulated over time, the showcase of incompetence in response to COVID-19 is profoundly concerning.^5^ From the chronic and consistent shortage of medical supplies to the moral crossroad of deciding who should be given a ventilator,^6^ the lack of disaster readiness has effectively caused health organizations and government agencies to regress into institutions of despair.

Worldwide, the lack of adequate response and emergency management in governments amid COVID-19 has upended the lives and livelihoods of individuals, has all but destroyed gross domestic products (GDPs), and has created avoidable vulnerabilities and ‘ground zeroes’ across continents.^5^ Despite the bleak situation, incompetence in the face of any disaster is not a permanent trait; instead, a more transient position improves with preemptive thinking and well-thought disaster preparation, such as a disaster readiness mindset. Disaster readiness is a state in which individuals or governments have both the plan, resources, and psychological prowess needed to mitigate or offset the potential adverse effects of disasters. This mindset involves the foresight and capability to effectively mitigate, prepare, respond, and recover amid and beyond disasters promptly (Fig. 1).

**Fig. 1.**
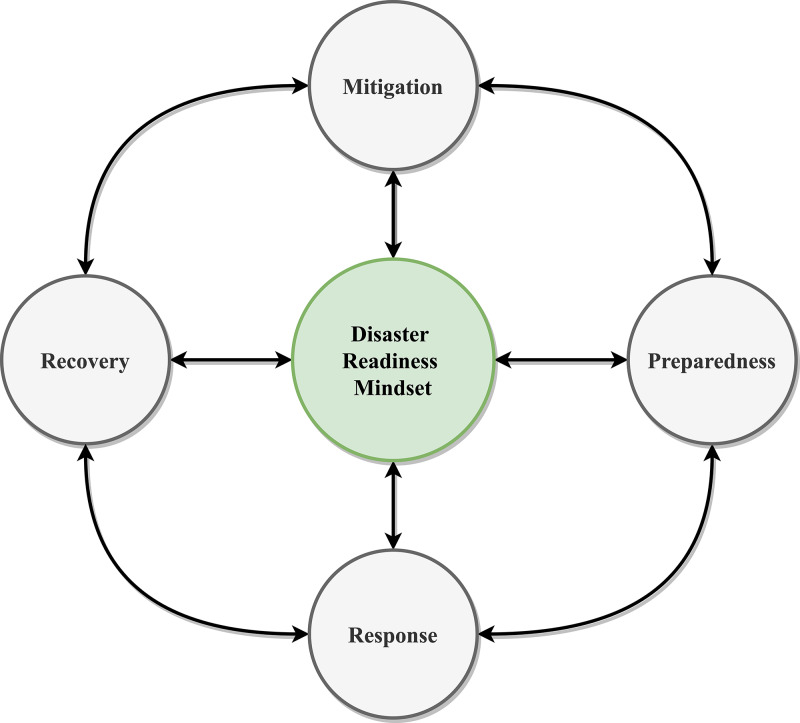
A schematic model of disaster readiness mindset.

Overall, a disaster readiness mindset is a preemptive, proactive, and people-centered philosophy that individuals and organizations need to ensure survival during and thriving after disaster outbreaks with tamed fear and uncertainty. A disaster readiness mindset may also be what is needed for organizations to mitigate effectively, prepare for, respond to, and recover from potential adverse impacts of disasters. It has the potential to help key stakeholders take into consideration most, if not all, of the factors that determine how disasters disappear in news feeds and appear in textbooks. With a disaster readiness mindset, insights from preemptive efforts and numerous disaster mitigation solutions in place, disasters can be more easily predicted, predicted, and controlled.

Ultimately, the disaster readiness mindset entails the following: (1) the foresight to develop a comprehensive disaster readiness plan that can help individuals and governments better cope with disasters cost-effectively; (2) the ability to locate and secure critical resources before a disaster that are needed for the society to survive and possibly thrive despite the adverse effects of the potentially catastrophic events; and (3) the agility, mobility, and flexibility required to execute the plan and deliver optimal results in an evolving situation. Finland, for instance, owing partly to the country’s ingrained awe towards its extensively bordered neighbor, arguably has the world’s most well-prepared and resource-abundant bunker systems.^7^ In the event of urgent disasters, these bunkers can host all of Finland’s population and protect its people from natural or human-made disasters. The bunkers are 18 m underground, span >124 miles, and can facilitate population-wide contamination mitigation procedures (eg, showers in the event of a chemical attack). They are equipped with food, bedding, medical facilities and supplies, and a wide selection of evening entertainment.^7^ In the wake of COVID-19, by repurposing resources, Finland is among the few countries reportedly unaffected by a shortage of medical supplies due to its disaster readiness mindset.^8^

The example of Finland shows that disaster readiness mindset can give a government the ability to identify antecedents to potential disasters, detail the potential risks a disaster might engender, and correspond with proactive strategies that either offset or minimize those risks. Disasters are a fact of life that can be prepared for but not avoided. Health organizations and government agencies across the world must develop a disaster readiness mindset so that even though COVID-19 is mutating^9^ and the looming possibility of future pandemics is constant,^10^ the damage of these disasters can become better predicted, prevented, and controlled.

## References

[1] Ricciardi A , Palmer ME , Yan ND. Should biological invasions be managed as natural disasters? BioScience 2011;61:312–317.

[2] Adger WN , Hughes TP , Folke C , Carpenter SR , Rockström J. Social-ecological resilience to coastal disasters. Science 2005;309:1036. 16099974 10.1126/science.1112122

[3] Su Z , McDonnell D , Ahmad J , et al. Time to stop the use of ‘Wuhan virus’, ‘China virus’ or ‘Chinese virus’ across the scientific community. BMJ Glob Health 2020;5(9):e003746. 10.1136/bmjgh-2020-003746 PMC7476418 32895218

[4] COVID-19 and racism—a double edged dagger. Lancet Diabetes Endocrinol 2020;8:649. 10.1016/S2213-8587(20)30243-6 PMC7351384 32659215

[5] Renda A , Castro R. Towards stronger EU governance of health threats after the COVID-19 pandemic. Eur J Risk Regul 2020;11:273–282.

[6] White DB , Lo B. A framework for rationing ventilators and critical care beds during the covid-19 pandemic. JAMA 2020;323:1773–1774. 32219367 10.1001/jama.2020.5046

[7] Grove T. Beneath Helsinki, Finns prepare for Russian threat. *The Wall Street Journal*. July 14, 2017.

[8] Anderson C , Libell HP . Finland, ‘Prepper nation of the Nordics,’ Isn’t worried about masks. *New York Times*. April 5, 2020.

[9] Grubaugh ND , Hanage WP , Rasmussen AL. Making sense of mutation: What D614G means for the COVID-19 pandemic remains unclear. Cell 2020;182:794–795. 32697970 10.1016/j.cell.2020.06.040 PMC7332445

[10] Honigsbaum M. Disease X and other unknowns. Lancet 2019; 393:1496–1497. 30983581 10.1016/S0140-6736(19)30803-7 PMC7136972

